# N-acetyl-cysteine prevents age-related hearing loss and the progressive loss of inner hair cells in γ-glutamyl transferase 1 deficient mice

**DOI:** 10.18632/aging.100927

**Published:** 2016-03-14

**Authors:** Dalian Ding, Haiyan Jiang, Guang-Di Chen, Chantal Longo-Guess, Vijaya Prakash Krishnan Muthaiah, Cong Tian, Adam Sheppard, Richard Salvi, Kenneth R. Johnson

**Affiliations:** ^1^ Center for Hearing and Deafness, University at Buffalo, Buffalo, NY 14214, USA; ^2^ The Jackson Laboratory, Bar Harbor, ME 04609, USA

**Keywords:** Inner hair cell loss, dwarf grey Ggt1^dwg/dwg^, otoacoustic emission, compound action potential, vestibular dysfunction, auditory brainstem response, glutathione, N-acetyl-L-cysteine

## Abstract

Genetic factors combined with oxidative stress are major determinants of age-related hearing loss (ARHL), one of the most prevalent disorders of the elderly. Dwarf grey mice, *Ggt1^dwg/dwg^*, are homozygous for a loss of function mutation of the γ-glutamyl transferase 1 gene, which encodes an important antioxidant enzyme critical for the resynthesis of glutathione (GSH). Since GSH reduces oxidative damage, we hypothesized that *Ggt1^dwg/dwg^* mice would be susceptible to ARHL. Surprisingly, otoacoustic emissions and cochlear microphonic potentials, which reflect cochlear outer hair cell (OHC) function, were largely unaffected in mutant mice, whereas auditory brainstem responses and the compound action potential were grossly abnormal. These functional deficits were associated with an unusual and selective loss of inner hair cells (IHC), but retention of OHC and auditory nerve fibers. Remarkably, hearing deficits and IHC loss were completely prevented by N-acetyl-L-cysteine, which induces *de novo* synthesis of GSH; however, hearing deficits and IHC loss reappeared when treatment was discontinued. Ggt1*^dwg/dwg^*mice represent an important new model for investigating ARHL, therapeutic interventions, and understanding the perceptual and electrophysiological consequences of sensory deprivation caused by the loss of sensory input exclusively from IHC.

## INTRODUCTION

Age-related hearing loss (ARHL) is one of the most prevalent disorders of the elderly. Nearly 25% of Americans 65-74 years of age have disabling hearing loss; this figure rises to 50% among those 75 or older (http://www.nidcd.nih.gov/health/statistics/Pages/quick.aspx). Severe hearing loss is a debilitating condition that can lead to communication difficulties, social isolation, depression and cognitive decline [1, 2]. Not surprisingly, susceptibility to ARHL is a complex, multifactorial disorder involving genetic, environmental, health and nutritional factors [3–6]. At the molecular level, the most overarching and widespread theory of aging involves oxidative stress arising from reactive oxygen or nitrogen species [7, 8]. Human heritability studies suggest that 25-75% of the variance in ARHL has a genetic basis [9, 10]. Allelic variations and mutations in human oxidative stress genes, such as glutathione S-transferase and N-acetyltransferase, appear to contribute to ARHL susceptibility [11, 12]; however, others have failed to find such an association [13] possibly due to heterogeneity of the sample population. Animal studies of ARHL conducted with inbred mice circumvent many of the problems associated with genetically heterogeneous human samples. Murine models have proved highly effective in isolating genes that regulate the progression of ARHL [14–17] and have also proved useful in identifying therapeutic interventions to suppress ARHL [18, 19].

In the current study, we discovered that dwarf grey mutant mice (*Ggt1^dwg/dwg^*, hereafter *dwg/dwg*) provide an important new animal model to study the development of ARHL. The *dwg/dwg* mice are homozygous for a spontaneous loss of function mutation of the *Ggt1* gene [20], which codes for γ -glutamyl transferase 1 (GGT1), a cell surface glycoprotein involved in the transfer of the glutamyl moiety of glutathione (GSH) to an acceptor molecule. Intracellular GSH, resynthesized through the γ-glutamyl cycle, is critical for protecting cells from oxidative stress. Loss of GGT1 leads to the depletion of intracellular GSH resulting in greater oxidative stress due to the depletion of this important antioxidant. Since GSH plays an important role in reducing oxidative stress in the inner ear, we hypothesized that *dwg/dwg* mice would be hearing impaired or they would develop ARHL and cochlear pathologies. To test this hypothesis, we evaluated auditory function as well as cochlear and vestibular histopathologies in WT (*+/+*), *Ggt1 ^dwg/+^* (*dwg/+*) and *dwg/dwg* mice from 1 to 9 months of age. Consistent with our prediction, *dwg/dwg* mice developed ARHL; however, the hearing loss was unexpectedly associated with a highly selective loss of IHC, an unusual and extremely rare type of cochlear pathology. Since *dwg/dwg* mutants are deficient in intracellular GSH [21] and susceptible to oxidative stress, we hypothesized that n-acetyl-L-cysteine (NAC), which promotes the *de novo* synthesis of GSH [22, 23], would prevent ARHL and IHC loss while receiving treatment, but that ARHL and hair cell loss would reappear when NAC treatment was discontinued.

## RESULTS

### Phenotype of *dwg/dwg* mice

The dwarf gray (*dwg*) mutation is a spontaneous 13 bp deletion in exon 7 of the *Ggt1* gene, which causes a frameshift and premature stop codon [20]. Consistent with previous reports, *dwg*/*dwg* mice [21, 24], like mice homozygous for the targeted *Ggt1^tm1Zuk^*[25–28] and mice homozygous for the ENU-induced *Ggt1^enu1^* [29, 30], exhibit dwarfism, skeletal abnormalities, diluted pigmentation, cataracts, and reduced fertility. The mutations differ, however, in the effects they have on life span. Mice homozygous for the *Ggt1^tm1Zuk^* (on a mixed 129SvE-C57BL/6J strain background) generally die by 10-18 weeks of age with only 10% survival at 25 weeks. Mice homozygous for the ENU-induced *Ggt1^enu1^* (on a C57BL/6J strain background) also have a shortened life span (50% survival at 25 weeks). In contrast, mice homozygous for the *Ggt1^dwg^* mutation *(on an inbred strain with undefined mixed stock origins)* live beyond one year of age with similar survival to controls. Because all three *Ggt1* mutations are thought to result in a loss of GGT1 function, the differences in longevity are likely influenced by the differences in strain backgrounds. The long life span of *dwg/dwg* mice allows for the assessment of late onset, age-related effects of GGT1 deficiency.

### ABR thresholds increase with age in *^dwg/dwg^* mice

**Figure 1:**
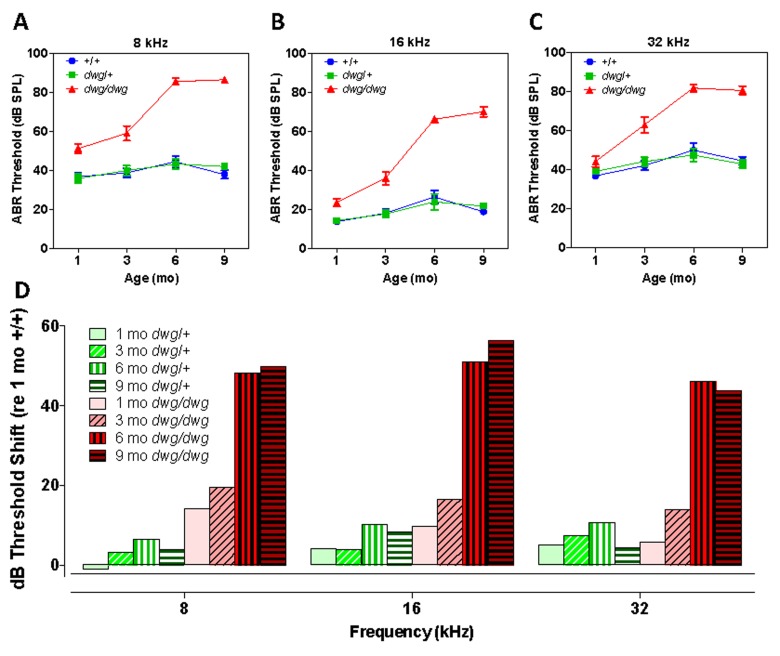
*Dwg/dwg* mice develop ARHL Mean (+/−SEM) auditory brainstem response (ABR) thresholds of *+/+, +/dwg and dwg/dwg* mice at 1, 3, 6 and 9 months of age at (**A**) 8 kHz, (**B**) 16 kHz and (**C**) 32 kHz. (**D**) Mean (+/−SEM) ABR threshold shifts of *+/dwg* and *dwg/dwg* mice relative to *+/+* mice at 1, 3, 6 and 9 months of age at 8, 16 and 32 kHz (n= 29, 29, 26 and 15 for *dwg/dwg* mice at 1, 3, 6 and 9 months of age; n = 11, 12, 12, 12 for *+/+* mice at 1, 3, 6 and 9 months of age and n = 12, 12, 12 and 12 for +/dwg mice at 1, 3, 6 and 9 months of ages).

To determine if mice heterozygous or homozygous for the *dwg* mutation would develop ARHL at an accelerated rate compared to *+/+* mice, ABR thresholds were measured at 1, 3, 6 and 9 months of age at 8, 16 and 32 kHz (Fig. 1A-C). At 8 and 16 kHz ABR thresholds in the *+/+* and *dwg/+* groups showed a slight to modest increase between 1 and 9 months of age (Fig. 1A-B). In contrast, 8 and 16 kHz thresholds in the *dwg/dwg* mice were already higher than the other groups at 1 and 3 months of age, evidence of early-onset hearing loss. Between 3 and 9 months of age, thresholds in the *dwg/dwg* increased substantially at all three test frequencies.

At 8 kHz, mean thresholds in the *+/+* and *dwg/+* groups ranged from ~36 to ~44 dB SPL at 1 month to 9 months of age whereas ABR thresholds in the *dwg/dwg* group were consistently higher than the *dwg/+* and *+/+* groups at all ages tested (Fig. 1A). Thresholds were nearly identical for the *+/dwg* and *+/+* groups at each individual age; however, there was a slight increase in threshold between 1 and 6-9 months of age. There was a significant (two-way ANOVA) effect of genotype (F= 163.0, p<0.0001, DF: 2, 182), age (F=19.47, p<0.0001, DF: 3, 182) and an age-genotype interaction (F=10.68, p<0.0001, DF: 6, 182). There was no significant difference in thresholds between *dwg/+* and *+/+* genotype at 1, 3, 6 or 9 months of age whereas the *dwg/dwg* mice had significantly higher thresholds than the other two genotypes at all four ages (Bonferroni post-test, p<0.01).

At 16 kHz, mean thresholds in the *+/+* and *dwg/+* groups ranged from ~14 to 26 dB SPL between 1 and 9 months of age whereas ABR thresholds in the *dwg/dwg* group were consistently higher than those in the *dwg/+* and *+/+* groups at all ages tested (Fig. 1B). Thresholds were nearly the same for the *dwg/+* and *+/+* groups at each of the individual ages; however, there was a slight increase in threshold between 1 and 6-9 months of age. There was a significant (two-way ANOVA) effect of genotype (F= 171.1, p<0.0001, DF: 2, 182), age (F=38.43, p<0.0001, DF: 3, 182) and an age-genotype interaction (F=17.04, p<0.0001, DF: 6, 182). There was no significant difference in thresholds between *dwg/+* and *+/+* genotype at 1, 3, 6 or 9 months of age. However, the *dwg/dwg* mice had significantly higher thresholds than the other two genotypes at 3, 6 and 9 months of age (Bonferroni post-test, p<0.05); the *dwg/dwg* group also had significantly higher thresholds than the *+/+* group at 1 month of age (Bonferroni post-test, p<0.05).

At 32 kHz, mean thresholds in the *+/+* and *dwg/+* groups ranged from ~36 to 50 dB SPL between 1 and 9 months of age. In contrast, the ABR thresholds in the *dwg/dwg* group were slightly higher than in the *dwg/+* and *+/+* groups at 1 and 3 months of age and substantially higher at 6 and 9 months (Fig. 1C).

Thresholds for the *dwg/+* and +/+ groups were very similar at each of the individual ages; however there was a modest increase in threshold between 1 and 6-9 months of age. There was a significant (two-way ANOVA) effect of genotype (F= 83.42, p<0.0001, DF: 2, 182), age (F=19.88, p<0.0001, DF: 3, 182) and an age-genotype interaction (F=7.22, p<0.0001, DF: 6, 182). There was no significant difference in thresholds between *dwg/+* and *+/+* genotype at 1, 3, 6 and 9 months of age; however, the *dwg/dwg* mice had significantly higher thresholds than *dwg/+* and *+/+* mice at 3, 6 and 9 months of age (Bonferroni post-test, p<0.001). ABR thresholds of mice sorted by age, genotype, and sex are shown in Table 1.

**Table 1:** Average ABR thresholds (dB SPL) of mice sorted by age, genotype, and sex

		number	average	8 kHz thresholds		16 kHz thresholds		32 kHz thresholds	
*Ggt1* genotype	sex	tested	weight (g)	Mean	Std Dev	Mean	Std Dev	Mean	Std Dev
**1 month test age**									
*+/+*	female	6	16.3	38.3	7.5	13.3	2.6	38.3	5.2
*+/+*	male	5	20.0	35.0	5.0	14.0	4.2	35.0	3.5
*dwg/+*	female	5	16.4	37.0	11.5	13.0	5.7	38.0	4.5
*dwg/+*	male	7	20.3	35.0	6.5	15.0	2.9	40.0	5.8
*dwg/dwg*	female	15	9.5	50.3	13.3	21.7	8.2	43.3	12.2
*dwg/dwg*	male	14	10.4	52.1	14.8	25.4	14.5	45.0	17.8
**3 month test age**									
*+/+*	female	7	24.7	36.4	8.5	16.4	6.9	40.7	7.9
*+/+*	male	5	26.8	42.0	6.7	20.0	10.0	44.0	8.9
*dwg/+*	female	5	25.2	37.0	8.4	14.0	4.2	43.0	8.4
*dwg/+*	male	7	29.0	42.1	8.6	20.0	7.1	45.0	7.6
*dwg/dwg*	female	16	15.8	60.3	22.3	33.8	20.0	62.8	22.3
*dwg/dwg*	male	13	15.6	57.7	18.4	38.5	16.1	63.1	22.5
**6 month test age**									
*+/+*	female	6	34.2	42.5	5.2	20.0	6.3	45.0	8.9
*+/+*	male	6	37.5	40.0	5.5	22.5	5.2	45.8	3.8
*dwg/+*	female	5	32.2	40.0	3.5	18.0	7.6	45.0	7.9
*dwg/+*	male	7	39.4	40.7	4.5	22.1	5.7	43.6	6.3
*dwg/dwg*	female	13	17.2	85.8	8.4	66.9	7.8	83.1	10.9
*dwg/dwg*	male	13	17.8	85.8	8.9	65.4	8.5	80.8	7.3
**9 month test age**									
*+/+*	female	7	38.3	37.1	4.9	18.6	4.8	48.6	6.3
*+/+*	male	5	39.6	39.0	10.8	19.0	4.2	39.0	4.2
*dwg/+*	female	5	36.8	39.0	4.2	18.0	5.7	41.0	8.2
*dwg/+*	male	7	40.1	44.3	4.5	24.3	3.5	44.3	6.1
*dwg/dwg*	female	10	18.8	87.0	5.9	70.0	10.8	82.5	9.8
*dwg/dwg*	male	5	19.6	86.0	4.2	70.0	8.7	77.0	2.7

Standard deviations (Std Dev) of the means are shown to indicate the extent of threshold variation observed among mice within each class.

To illustrate the age-related progression of hearing loss in the *dwg/+* and *dwg/dwg* mice, the ABR thresholds in these two genotypes were compared to the thresholds in 1-month-old *+/+* mice to determine the amount of threshold shift at each frequency and age. At 8 kHz, the average threshold shifts in the *dwg/+* mice were −1, 3.2, 6.5 and 3.8 dB at 1, 3, 6 and 9 months respectively (Fig. 1D). In contrast, there was significant increase in 8 kHz thresholds in the *dwg/dwg* mice; at ages 1, 3, 6 and 9 months the mean threshold shifts were 14.2, 19.4, 48.2 and 50 dB respectively. At 16 kHz, the mean threshold shifts in the *dwg/+* group were 4.2, 3.9, 10.1 and 8.2 dB at 1, 3, 6 and 9 months of age versus 9.6, 16.4, 51.1 and 56.4 dB in the *dwg/dwg* group at these ages. At 32 kHz, the mean threshold shifts in the *dwg/+* group were 5.2, 7.4, 10.7 and 4.4 dB at 1, 3, 6 and 9 months of age while in the *dwg/dwg* group the threshold shifts were 5.7, 13.8, 46.0 and 43.9 dB respectively at these ages. Taken together, these results indicate that one normal copy of the *Ggt1* gene is sufficient to delay or prevent the age-related decline in hearing over the 1-9 month period.

### IHC lesions increase with age in *dwg/dwg* mice

**Figure 2:**
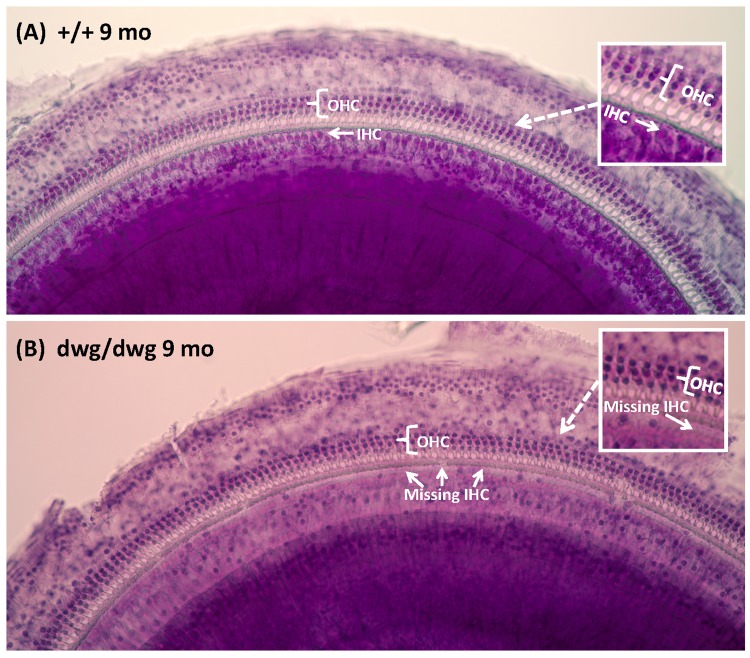
Surface preparations showing missing IHC in 9-month-old *dwg/dwg* mice Representative surface preparation stained with hematoxylin from the middle of the cochlea of 9-monthold *+/+* mice and *dwg/dwg* mice. (**A**) Organ of Corti of *+/+* mouse with three orderly rows of outer hair cells (OHC) and one row of inner hair cells (IHC); inset shows higher magnification view of three rows of OHC and one row of IHC. (**B**) Organ of Corti from *dwg/dwg* mouse with three rows of OHC; most IHC were missing; inset shows higher magnification view of organ of Corti with 3 rows of OHC and missing IHC.

**Figure 3:**
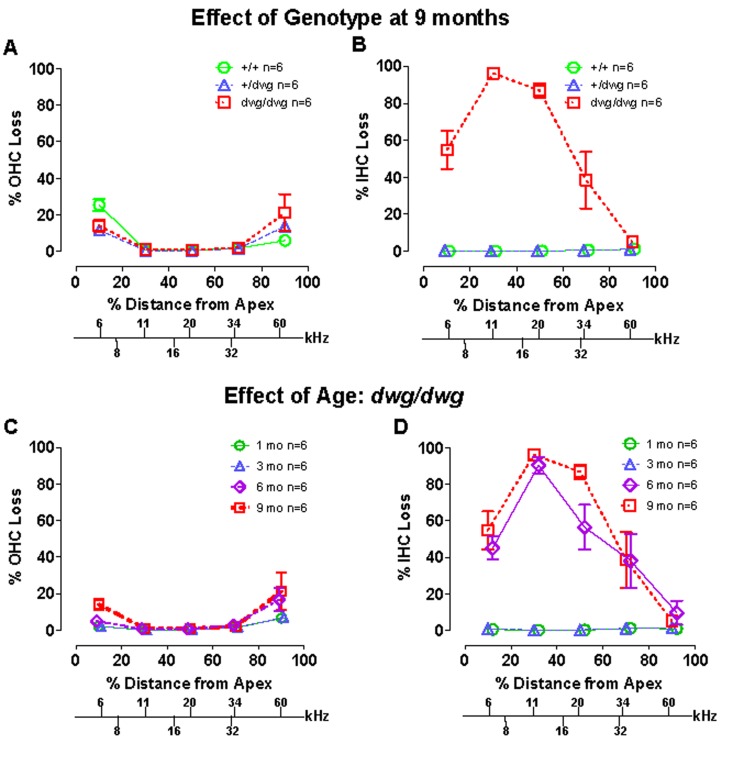
*Dwg/dwg* mice show large IHC loss in the apical 70% of cochlea at 6-9 months of age (**A**–**B**) Mean % OHC and IHC loss (+/− SEM, n=6) as a function of % distance from the apex of the cochlea for 9 month old *+/+*, *+/dwg* and *dwg/dwg* mice. Frequency-place map for mouse cochlea is shown on abscissa. Note large IHC loss in the apical 70% of the cochlea and little OHC loss in *dwg/dwg* mice and also minimal OHC and IHC loss in *+/+* and *+/dwg* mice. (**C**–**D**) Mean % OHC and IHC loss in *dwg/dwg* mice (20% distance intervals, +/− SEM, n=6) as a function of % distance from the apex of the cochlea in 1, 3, 6 and 9 month old *dwg/dwg* mice. Frequency-place map for mouse cochlea shown on abscissa [124].

Most cases of hearing loss are associated with hair cell loss that begins in the basal high-frequency region of the cochlea and spreads towards the apex with increasing age, and in the majority of cases OHC loss occurs prior to IHC loss [14, 31, 32]. However, in *dwg/dwg* mice, the onset of hearing loss occurred earlier at 8 and 16 kHz than 32 kHz. This unconventional hearing loss profile suggested that the location and progression of cochlear pathology might proceed along a different trajectory in *dwg/dwg* mice. To test this hypothesis, we prepared individual cochleograms for the three genotypes and computed the percent OHC and IHC loss in 20% intervals along the length of the cochlea. Figure 2 shows photomicrographs from the middle turn of the cochlea of representative +/+ and *dwg/dwg* mice. Three orderly rows of OHC and one row of IHC are present in the *+/+* mouse (Fig. 2A); the higher magnification inset shows the nuclei in the three rows of OHC and one row of IHC. Three orderly rows of OHC were also present in the middle turn of *dwg/dwg* mice; however, the vast majority of IHC were missing (Fig. 2B). This is illustrated more clearly in the higher magnification inset that shows the nuclei in the three rows of OHC, but absent nuclei in the region normally occupied by IHC. To quantify the hair cell lesions, mean cochleograms were prepared from 9-month-old *+/+*, *dwg/+* and *dwg/dwg* mice. At 9 months of age, there was minimal OHC loss in all the genotypes except for very mild losses (<25%) in the most apical and basal regions (Fig. 3A); the slight differences between genotypes were not statistically significant. There was also little IHC loss in the *+/+* and *dwg/+* mice (Fig. 3B). In contrast, *dwg/dwg* mice had severe IHC losses: 96% in the region 30% from the apex of the cochlea, corresponding to roughly 11 kHz on the tonotopic map, and 39% in the region 70% from the apex, corresponding to 34 kHz [33]. While *+/+* and *dwg/+* genotypes showed remarkably little hair cell loss at 9 months of age, there was a massive loss of IHC in the apical two-thirds of the cochlea in *dwg/dwg* mice, but no OHC loss.

By 3 months of age, ABR thresholds were higher in mutant *dwg/dwg* mice than in non-mutant *dwg*/+ and +/+ mice (Fig. 1A-C) suggesting that a modest IHC pathology might already be present at an early age. In addition, the rapid increase in threshold between 3 and 6 months suggested that IHC pathology would dramatically escalate during this time. To test these hypotheses, hair cell lesions were measured between 1 and 9 months of age in *dwg/dwg* mice. There was a slight, but insignificant increase in OHC loss between 3 and 9 months of age; this minor increase was confined to the most apical and basal regions of the cochlea (Fig. 3C).

Virtually all IHC were present at 1 and 3 months of age in *dwg/dwg* mice (Fig. 3D). However, a massive increase in IHC loss occurred between 3 and 6 months of age; this was followed by a modest increase between 6 and 9 months of age. There was a statistically significant increase in IHC loss with age (One-way ANOVA, F=8.02, 3, 16 DF, p<0.001), and statistically significant differences were observed between 1 and 6 months, 1 and 9 months, 3 and 6 months, and 3 and 9 months of age (Tukey's post-hoc comparison, p<0.05). The rapid increase in IHC loss between 3 and 6 months of age is consistent with the rapid rise in ABR thresholds at this time. On the other hand, there was no evidence of IHC or OHC loss in *dwg/dwg* mice at 3 months of age that would account for their higher thresholds relative to +/+ and *dwg*/+ mice (Fig. 1).

**Figure 4:**
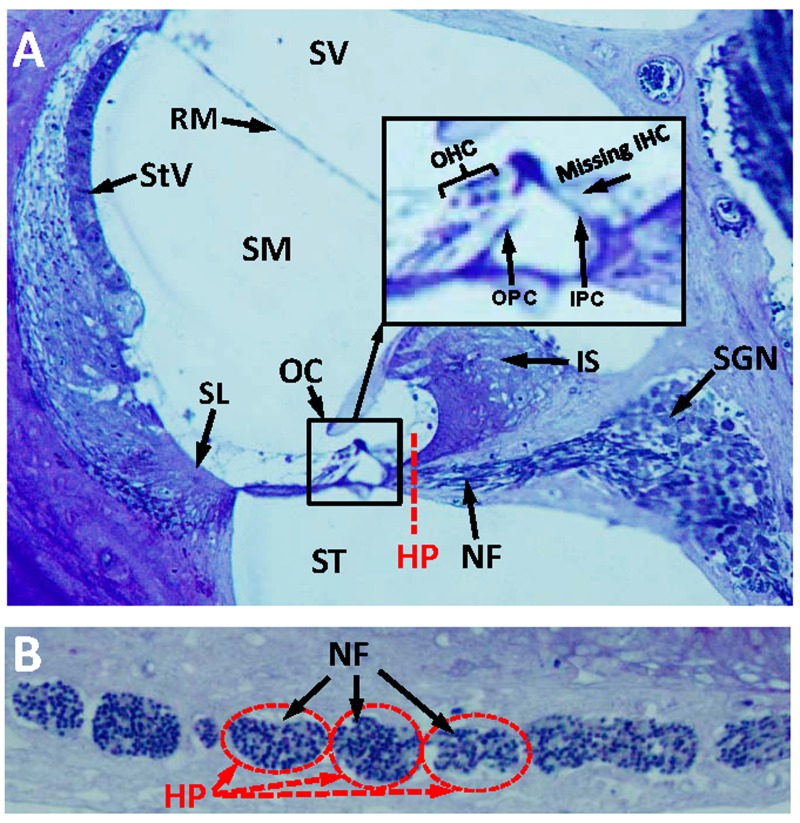
Radial section of cochlea showing missing IHC, but intact nerve fibers in the habenula perforata (**A**) Toluidine stained radial section (3 μM) from the middle turn of an Epon embedded cochlea from a 9-month-old *dwg/dwg* mouse stained with toluidine blue. Stria vascularis (StV), scala vestibuli (SV), scala media (SM),scala tympani (ST), organ of Corti (OC), nerve fibers (NF) and spiral ganglion neurons (SGN). Inset shows higher magnification view of OC with three rows of OHC, inner pillar cell (IPC), outer pillar cell (OPC), but missing IHC. Dashed red line shows approximate plane of section through the habenula perforata (HP). (**B**) Toluidine stained section (3 μM) tangential to the habenula perforata. Habenular openings densely packed with nerve fibers.

The discrepancy between elevated thresholds and intact hair cells at 1-3 months of age raised the possibility that other neurodegenerative changes besides IHC loss might be occurring in *dwg/dwg* mice such as of loss of auditory nerve fibers [34–36] or damage to non-sensory cells of the cochlea. To test for other significant morphological changes, we evaluated 3 μm radial sections of the cochleae by light microscopy in 9-month-old *dwg/dwg* mice, an age at which there is major IHC loss and hearing loss. At a gross level, the overall structural integrity of the cochlea looked remarkably normal with no obvious damage to the spiral ligament (SL), stria vascularis (StV), Reissner's membrane (RM), inner sulcus (IS), tectorial membrane, or organ of Corti (OC) (Fig. 4A). Rosenthal's canal was filled with spiral ganglion neurons (SGN) and the canal leading to the habenula perforata (HP) and organ of Corti was filled with nerve fibers (NF). A high magnification view (inset Fig. 4A) of the OC shows the tunnel of Corti with inner pillar cells (IPC), outer pillar cells (OPC), and three rows of OHC, but absent IHC. Sections tangential to the HP revealed a dense packing of NF coursing through the openings in the osseous spiral lamina. Despite the massive loss of IHC, the NF and SGN looked remarkably normal at this level of light microscopic analysis. Because cochlear pathology appears to be restricted to IHC, the discrepancy between elevated ABR thresholds and intact IHC at 3 months of age suggests that some IHC function is lost prior to cellular degeneration.

### Cochlear function

The ABR, which reflects downstream activity generated in the auditory brainstem is a non-specific and indirect measure of cochlear dysfunction. To gain insights on the functional status of the cochlea, which appeared structurally normal we performed electrophysiological tests of cochlear function.

**Figure 5:**
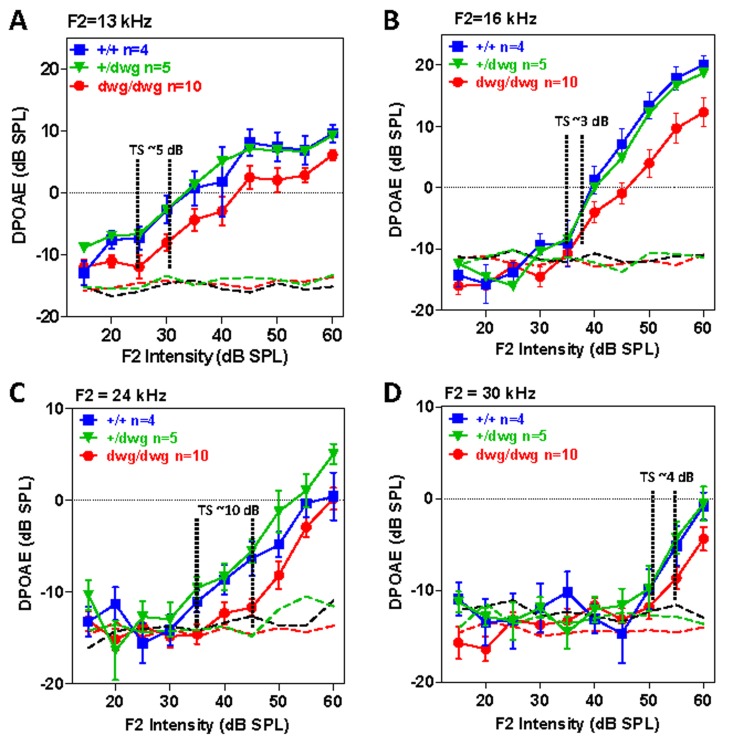
DPOAE in 6-month-old *dwg/dwg* mice present, but slightly reduced relative to *+/+* and *dwg/+* mice Mean (+/− SEM, n shown in panels) distortion product otoacoustic emission (DPOAE) input/output functions from 6-month-old *+/+*, *+/dwg* and *dwg/dwg* mice at F2 frequencies of 13, 16, 24 and 30 kHz (thin dashed lines shows noise floor of system). DPOAE amplitudes for all three groups are well above the noise floor; DPOAE amplitudes for dwg/dwg mice are consistently smaller than those from *+/+* and *+/dwg* mice.

DPOAE provide a noninvasive method for assessing the nonlinear, electromotile response of OHC in collaboration with the endolymphatic potential. We measured the DPOAE I/O functions in different frequency regions in a cohort of 6-month-old *+/+*, *dwg/+* and *dwg/dwg* mice. DPOAE amplitudes increased with F2 intensity and responses were well above the noise floor in all three genotypes (Fig. 5A-B). While DPOAE I/O functions were nearly identical in *+/+* and *dwg/+* mice, the amplitudes were slightly lower (~2-8 dB) and the I/O functions shifted to right in *dwg/dwg* mice. We attempted to estimate the DPOAE “threshold shifts” by drawing vertical lines through the points on each I/O functions at which responses just started to rise above the noise floor (the threshold) and then measured the horizontal distance between the “threshold” of *dwg/dwg* versus +/+ and *dwg*/+ mice (Fig. 5A-D). DPOAE “threshold shifts” were approximately 5, 3, 10 and 4 dB at F2 frequencies of 13, 16, 24 and 30 kHz. These results suggest that only a mild functional deficit exists in the DPOAE generators of *dwg/dwg* mice, possibly in the OHC and/or the stria vascularis that produces the endolymphatic potential or alternatively in middle ear anomalies.

**Figure 6:**
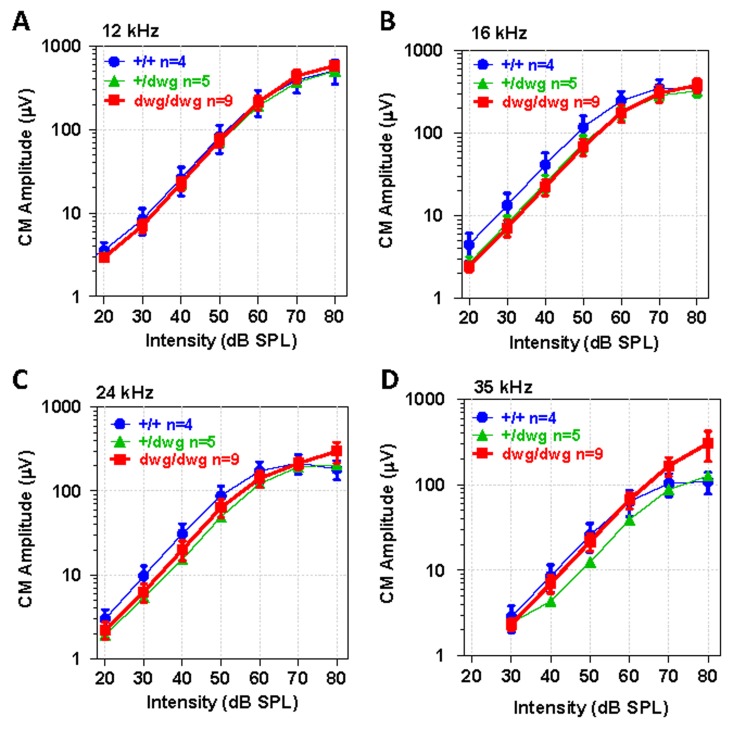
CM potential normal in 6-month-old *dwg/dwg* mice Mean (+/−SEM, n shown in panel) cochlear microphonic (CM) amplitude-intensity functions from 6-month-old *+/+*, *+/dwg* and *dwg/dwg* mice at 12, 16, 24 and 35 kHz. CM amplitudes increase with level and values plateau around 80 dB SPL.

To identify potential dysfunctions in the transduction apparatus of OHC, we recorded CM I/O functions from the round window of the same mice used for DPOAE testing. The CM recorded in this manner is dominated by activity from OHC located near the round window membrane in the high frequency region of the cochlea and is largely independent of IHC loss [37–40]. The CM I/O functions recorded from *dwg/dwg* mice were very similar to those measured in *+/+* and *dwg*/+ mice with relatively minor differences between the three genotypes at the four frequencies tested, 12, 16, 24 and 35 kHz (Fig. 6 A-D). These results indicate that the transduction apparatus located in the apical pole of OHC is functionally intact, findings that reinforce the view that the CM is predominantly generated by OHC and that IHC loss has little effect on CM amplitude [37, 38].

**Figure 7:**
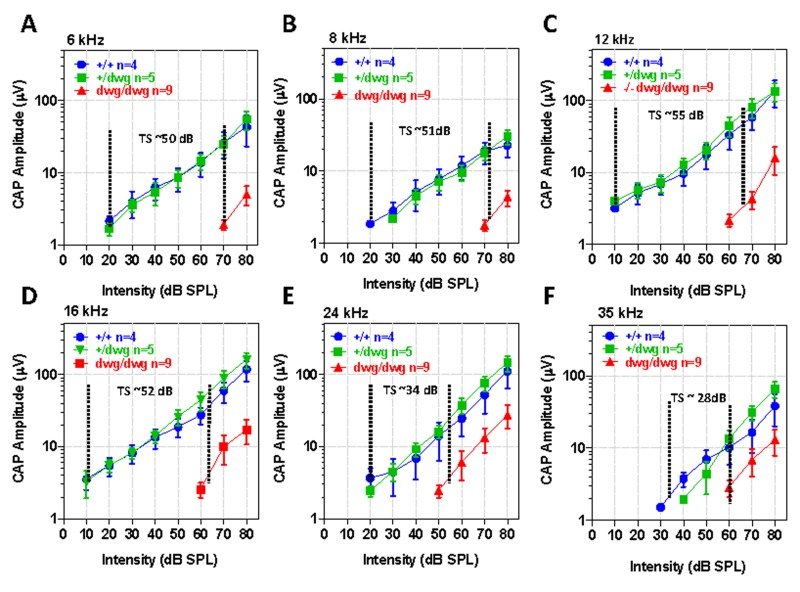
CAP amplitudes greatly reduced in *dwg/dwg* mice compared to *+/+* and *+/dwg* mice Mean (+/− SEM, n shown in panels) compound action potential (CAP) input/output functions at 6, 8, 12, 16, 24 and 35 kHz from 6-month-old *+/+*, *+/dwg* and *dwg/dwg* mice. CAP amplitudes in *dwg/dwg* mice are greatly reduced compared to *+/+* and *+/dwg* mice; amplitudes in *+/+* and *+/dwg* mice are similar. Horizontal dashed line and number indicates the approximate threshold shift (TS) in *dwg/dwg* mice compared to *+/+* mice.

Although DPOAE “thresholds” were elevated in *dwg/dwg* mice relative to the other genotypes, these shifts were considerably smaller than for the ABR. To identify additional functional deficits downstream of the OHC, we measured CAP I/O functions in the same cohort of mice used above. The CAP is predominantly generated by the synchronous onset response of type I auditory nerve fibers due to the release of excitatory neuro-transmitter from IHC. Therefore, we expected CAP amplitudes would be greatly reduced in *dwg/dwg* mice. The CAP I/O functions in *dwg/+* mice were nearly identical to *+/+* mice (Fig. 7A-F). In these two groups, the logarithm of CAP amplitude increased approximately linearly with dB SPL reaching levels exceeding 100 mV from 12-24 kHz. Near-threshold CAP responses of 2-3 mV were detected at levels as low as 10 dB SPL for some frequencies indicative of excellent sensitivity. The CAP I/O functions in the *dwg/dwg* mice were also linear on a log-log plot; however, the maximum amplitudes were greatly reduced compared with those of *+/+* and *dwg*/+ mice and amplitudes were typically less than 30 mV. We estimated the CAP “threshold shifts” by draw-ing vertical lines through the point on each I/O function at which responses just started to rise above the noise floor (the threshold) and then measured the horizontal distance between the threshold lines of *dwg/dwg* and +/+ mice (Fig. 7A-F). The CAP threshold shifts were approximately 50, 51, 55, 52, 34 and 28 dB at 6, 8, 12, 16, 24 and 35 kHz respectively, shifts slightly greater than for the ABR (Fig. 1). The CAP threshold shifts were greater at low frequencies than high frequencies, consistent with the location of IHC lesions, which were most severe in the 11-20 kHz region of the tonotopic map and minimal in the extreme base of the cochlea (Fig. 3).

### Vestibular dysfunction

**Figure 8:**
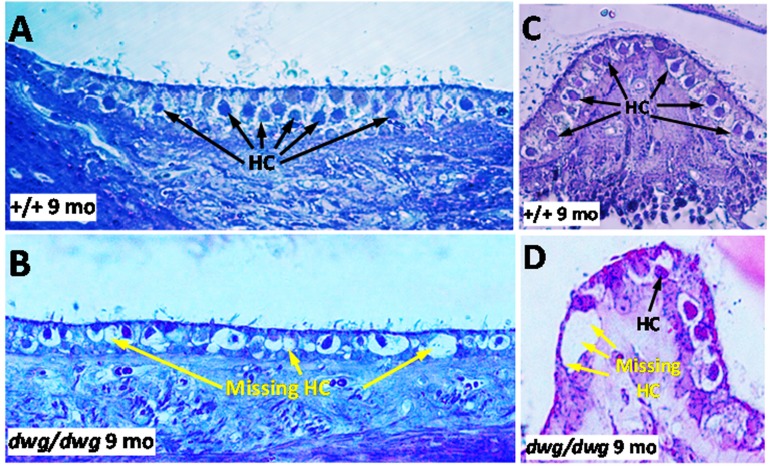
Epon embedded cross sections (3 μM) of the macula of the utricle stained with toluidine blue from a 9-month-old (**A**) *+/+* mouse and a (**B**) *dwg/dwg* mouse. Numerous hair cells (HC) surrounded by large translucent afferent synapses decorated the vestibular hair cells in the sensory epithelium of *+/+* mouse whereas many vestibular hair cells were missing and large vacuoles present in the vestibular epithelium of saccule of *dwg/dwg* mice. Epon embedded cross section (3 μM) of the crista of the ampulla stained with toluidine blue from a 9 month old (**C**) *+/+* mouse and (**D**) *dwg/dwg* mouse. Numerous hair cells (HC) surround the translucent afferent synapses in the sensory epithelium in *+/+* mouse whereas many HC were missing in the crista of *dwg/dwg* mice.

As *dwg/dwg* mice age, they begin to exhibit behaviors characteristic of inner ear vestibular dysfunction. Around 3 months of age mutant mice begin exhibiting a head bobbing behavior and between 4 and 5 months, they start to show noticeable circling behavior; both behaviors increased with age. Late onset circling behavior in *dwg/dwg* mice was reported previously, but was attributed to central nervous system dysfunction rather than vestibular damage [20]. To determine if head bobbing and circling were associated with vestibular histopathologies, we evaluated 3 μm radial sections of the utricle, saccule and crista ampullaris by light microscopy at 9 months of age when head bobbing and circling behaviors were severe in *dwg/dwg* mice. Figure 8A-B compares the status of vestibular hair cells in the macula of the utricle in *+/+* and *dwg/dwg* mice. In *+/+ mice*, the ovoid somas of the vestibular hair cells lined the surface of the sensory epithelium (Fig. 8A). In contrast, numerous vacuoles and open spaces were present along the surface of the utricular sensory epithelium in *dwg/dwg* mice (Fig. 8B). Within these spaces, the somas of the residual hair cells were shrunken and distorted. Figure 8C-D compares the condition of the hair cells in the crista ampullaris of *+/+* and *dwg/dwg* mice. Vestibular hair cell somas were aligned along the upper surface of the crista in *+/+* mice (Fig. 8C) whereas in *dwg/dwg* mice hair cells were missing and replaced by large vacuoles and open space (Fig. 8D).

### NAC prevents hearing and hair cell loss

**Figure 9:**
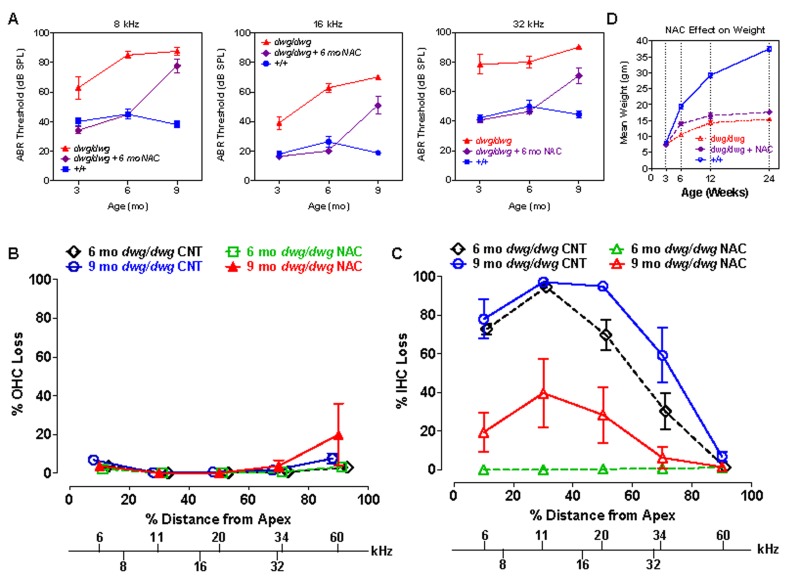
NAC supplementation for 6 months prevents hearing loss and IHC loss in *dwg/dwg* mice, but has little effect on body weight (**A**) Mean ABR thresholds at 8, 16 and 32 kHz in untreated *+/+* mice (n= 10, 10 and 10 for 3, 6 and 9 months respectively), untreated *dwg/dwg* mice (n=9, 9 and 2 for 3, 6 and 9 months respectively) and *dwg/dwg* mice treated with NAC for first 6 months and then untreated until 9 months (n= 15, 14 and 9 for 3, 6 and 9 months respectively). (**B**) Mean % OHC loss or (**C**) mean % IHC loss versus % distance from the apex of the cochlea in 6 month old untreated *dwg/dwg* control mice (n=6), 6 month old dwg/dwg mice (n=6) treated with NAC for 6 months (n=6), 9 month old *dwg/dwg* control mice (n=5) or 9 month old dwg/dwg mice (n=6) treated with NAC for 6 months and left untreated from 6-9 months. Frequency-place map for mouse cochlea is shown on abscissa [124]. (D) Average weight of untreated +/+ mice (n=6; 3-24 weeks), untreated *dwg/dwg* mice (n=8, 7, 5 and 5 for 3, 6, 12 and 24 weeks) and *dwg/dwg* mice treated with NAC for first 6 months (n=8, 7, 5 and 5 for 3, 6, 12 and 24 weeks).

Since NAC promotes the synthesis of glutathione and protects against several forms of hearing loss [22, 23, 41, 42], we treated *dwg/dwg* mice with NAC from 3 weeks to 6 months of age to determine if it would prevent hearing loss and hair cells loss. NAC treatment was discontinued at 6 months of age to ascertain if hearing and hair cells would deteriorate or remain stable at the 9-month test age. ABR thresholds at 8, 16 and 32 kHz in *+/+* mice were normal at 3 months of age and remained largely unchanged out to 9 months of age, whereas the thresholds of untreated *dwg/dwg* mice were considerably higher than *+/+* mice and also increased with age particularly at 8 and 16 kHz (Fig. 9A). In contrast, thresholds in the NAC-treated *dwg/dwg* group remained stable and were nearly identical to the thresholds in the *+/+* mice up to 6 months of age when treatment was discontinued. When tested again at 9 months of age (after 3 months without NAC supplementation) the mean thresholds of these mice had increased more than 25 dB. In addition, the NAC-treated *dwg/dwg* mice did not develop the late onset head bobbing and circling behaviors characteristic of untreated *dwg/dwg* mice, indicating that NAC treatment is effective in preserving inner ear vestibular as well as cochlear function.

Hair cell loss was evaluated at 6 and 9 months of age in: (1) untreated *dwg/dwg* mice, (2) *dwg/dwg* mice at 6 months of age that were treated with NAC since 3 weeks of age, and (3) *dwg/dwg* mice at 9 months that received the identical NAC treatment up to 6 months after which treatment was discontinued. OHC lesions were minimal in all of the groups (Fig. 9B) consistent with previous results (Fig. 3). IHC lesions at 6 and 9 months of age were extremely large in the apical two-thirds of the cochlea of untreated *dwg/dwg* mice and IHC lesions in this region increased with age (Fig. 9C). By contrast, there was no IHC loss at 6 months of age in the *dwg/dwg* group continuously treated with NAC. However, IHC lesions were observed (20-40% IHC loss) in the apical two-thirds of the cochlea of the group of 9-month-old *dwg/dwg* mice in which NAC treatment had been discontinued at 6 months.

These results indicate that continuous NAC treatment can completely prevent hearing loss and hair cell loss in *dwg/dwg* mice at least up to 6 months of age. Once treatment is discontinued hearing thresholds increase precipitously nearly catching up to the thresholds in the untreated group at 9 months of age. IHC loss also increases after cessation of NAC treatment; however, it is less severe (or slower to occur) than ABR threshold elevations. We conclude that NAC treatment is an effective otoprotective therapy that can largely abrogate the progressive inner ear dysfunction of dwg/*dwg* mutant mice. The NAC treatment results also indicate that the hearing loss and IHC loss of *dwg/dwg* mice are caused by cysteine and GSH deficiencies in the inner ear and are not secondary effects of dwarfism or other non-otic abnormalities.

As implied by the name “dwarf gray,” *dwg/dwg* mice are considerably smaller than *+/+ mice*, whereas *+/+* and *dwg*/+ mice have the same body weight and growth curve (Table 1). To determine if NAC treatment could reverse the effect of this mutation on stature, we measured the weights of *+/+*, untreated *dwg/dwg,* and *dwg/dwg* mice continuously supplemented with NAC from 3 weeks of age. The mean body weight of *+/+* mice increased between 3 and 24 weeks of age reaching a maximum of around 37.5 grams whereas body weight increased very slowly with age in untreated *dwg/dwg* mice reaching a maximum of 15.2 grams at 24 weeks of age (Fig. 9D). At 24 weeks of age, the mean body weight of the NAC-treated *dwg/dwg* mice was 17.6 grams, 2.2 grams higher than that of the untreated *dwg/dwg* mice, but substantially less than the 37.5 grams of the *+/+* mice. Statistical analysis of these results indicate that at 24 weeks of age the NAC-treated *dwg/dwg* mice were significantly heavier than untreated *dwg/dwg* mice (F=338.5, 2, 18 DF, p<0.0001, Newman-Keuls post-hoc analysis p<0.05) and that *+/+* mice were significantly heavier than NAC-treated and untreated *dwg/dwg* mice (p<0.05). Thus, NAC treatment decreased but did not completely prevent the effect of the *dwg* mutation on body size.

## DISCUSSION

We report here, for the first time, that GGT1 deficiency results in a progressive impairment of inner ear function. We show that *Ggt1^dwg/dwg^* (*dwg/dwg*) mice exhibit ARHL and balance defects caused by a massive loss of cochlear IHC and vestibular hair cells. The IHC lesions, which developed between 3 and 9 months of age in *dwg/dwg* mice, were most severe in the apical two-thirds of the cochlea; these lesions were associated with large CAP and ABR threshold shifts and a large reduction in CAP amplitude. Despite the massive loss of IHC, the OHC, support cells, stria vascularis and nerve fibers within the habenula perforata appeared intact, and CM amplitudes were normal while DPOAE amplitudes were only slightly reduced. NAC, which promotes the synthesis of GSH, prevented ABR threshold shifts and IHC losses; however, these effects were lost when NAC supplementation was discontinued.

### GGT, glutathione and oxidative stress

Metabolically active hair cells require high rates of mitochondrial ATP production; this results in the generation of reactive oxygen species (ROS) that can lead to oxidative damage if these highly toxic molecules are not inactivated by anti-oxidant enzymes such as GSH [28, 43]. GGT plays a key role in the resynthesis of GSH, and loss of GGT1 in *dwg/dwg* mice [21] and other *Ggt1* mutant mice [20, 25, 28] severely reduces intracellular GSH levels, which likely accounts for many of the pathologies in these mice. Many ototraumatic agents that induce oxidative stress also disrupt GSH homeostasis. Cochlear GSH levels are greatly reduced after cisplatin-induced ototoxicity [44, 45]. Depletion of GSH increases cisplatin and carboplatin ototoxicity [46–48], whereas treatments that increase GSH reduce ototoxicity [46, 49, 50]. Likewise treatments that increase cochlear GSH levels generally protect against noise-induced hearing loss [42, 51, 52], although this is not always the case [53, 54]. In contrast, conditions or genetic mutations that reduce GSH cause greater noise-induced hearing loss [17, 55].

### NAC Protection

There are conflicting reports in the literatures regarding the otoprotective capabilities of NAC. Some results suggest that NAC delays ARHL, noise-induced hearing loss and cisplatin or carboplatin ototoxicity [19, 56–59] while other studies suggest that NAC is ineffective [53, 54, 60]. However, NAC was highly effective in preventing hearing loss and IHC loss in our *dwg/dwg* mice with glutathione deficiency, consistent with reports in other GGT mutants showing that NAC prevents cataract formation and increases weight gain [21, 25, 27]. NAC's protective effect could be mediated in several ways. NAC has strong antioxidant properties and scavenges reactive oxygen species [61, 62]. NAC also upregulates reduced glutathione and glutathione peroxidase and suppresses the generation of reactive oxygen species by preventing lipid peroxidation [63]. In addition to inhibiting reactive oxygen species, NAC also inhibits the mTOR which in turn prevents senescence [64]. Drugs that suppress mTOR, such as rapamycin or metformin, have been shown to suppress cancer, type 2 diabetes and other age-related diseases [65–67]; however, their ability to prevent age-related or other forms of hearing loss such as those linked to diabetes remain largely untested [68, 69]. Interestingly, metformin, a drug that inhibits mTOR, attenuated aminoglycoside-induced hair cell loss in vitro, but it did not reduce hearing loss and hair cell damage in vivo [70].

### Cochlear GSH

GGT cleaves extracellular GSH (which cannot be directly transported into cells) into glutamate and cysteine-glycine, which is further degraded into cysteine and glycine [21, 25]. Cysteine, which is readily absorbed into cells, is the limiting amino acid in the resynthesis of GSH. Since GSH is one of the most important antioxidants, its cellular distribution could be one factor that accounts for the gradients of cochlear hair cell loss. GSH levels are significantly lower in guinea pig OHC in the base of the cochlea than in the apex; this GSH gradient may explain why OHC loss is greater in the base of the cochlea after a variety of ototraumatic insults [71]. However, in mice GSH levels are similar in the base and apex [72]; therefore, the absence of a clear GSH gradient in mice makes it difficult to explain the greater vulnerability of basal turn OHC in the mouse cochlea. GSH levels are significantly higher in IHC than OHC in guinea pigs [71, 73]; this could explain why IHC are more resistant to most ototraumatic agents than OHC. Conversely, the higher levels of GSH in IHC and the age-related increase in GSH in IHC, but not OHC [72] could be interpreted as evidence that IHC are normally under greater oxidative stress than OHC. Therefore, the inactivation of GGT in *dwg/dwg* mice would be expected to eliminate an important defense mechanism in IHC. While this is a possibility, it fails to account for the gradient of IHC loss, which is substantially greater in the apical half of the cochlea than the base.

Another hypothesis relevant to selective loss of IHC in *dwg/dwg* mice is that GGT1 deficiency leads to disrupted cysteine homeostasis [29] and that depletion of intracellular cysteine interferes with synaptic vesicle release by disrupting cysteine-string proteins which are present on IHC, but not OHC. Cysteine-string proteins, rich in cysteine residues, localize to synaptic vesicles tethered to IHC synaptic ribbons [74] and increase presynaptic calcium currents [75]. Importantly, cysteine-string proteins, members of the Dna/Hsp40 family, promote normal protein folding and exhibit anti-degenerative properties [76]. Mutations of cysteine-string proteins cause synaptic dysfunction and presynaptic neurodegeneration [77, 78]. Thus, GGT1-induced disturbances in cysteine could selectively damage IHC by affecting cysteine-string proteins. However, it is unclear at this time if there is a tonotopic gradient of these proteins that parallels the pattern of IHC loss in *dwg/dwg* mice.

### Role of cysteine in NAC supplementation

Continuous administration of NAC completely prevented hearing loss and IHC loss in *dwg/dwg* mice, but had only a moderate effect on body weight and did not fully prevent dwarfism (Fig. 9). This limited effect of NAC on body weight could reflect an early requirement of cysteine for normal body growth, but not for inner ear development. At weaning (~3 weeks), *dwg/dwg* mice are about 75-90% of the weight of WT littermate controls and by 6 weeks they are only about half the weight of WT controls (Fig. 6B; [21]). The early onset of dwarfism in mutant mice may be related to the importance of cysteine metabolism in the regulation of bone remodeling [79]. The later onset of hearing loss relative to reduced body size in *dwg* mutant mice suggests that hearing and vestibular impairments may have more to do with GSH levels and progressive oxidative damage than to cysteine metabolism. Brief GSH depletion reduces only cytosolic levels of GSH with little associated toxicity whereas long-term chronic GSH depletion reduces the mitochondrial GSH pool causing mitochondrial dysfunction with eventual adverse effects [28]. Thus, GGT1 deficient mice with chronic GSH depletion would be expected to exhibit a late onset, progressive deterioration of mitochondrial function leading to decreased ATP production, cell injury, and death of metabolically active cells. Because hair cells do not regenerate, their loss would accumulate with age.

### Models and mechanisms of IHC loss

The cochlea contains two types of sensory cells, OHC and IHC. The OHC imbue the cochlea with electromechanical feedback that amplifies the incoming sounds [80, 81]. Selective destruction of OHC abolishes DPOAE and results in approximately 50 dB hearing loss [82–84] indicating that OHC primarily act as cochlear amplifiers. Although there are far fewer IHC than OHC, IHC receive 90-95% of the auditory nerve fiber innervation and the auditory nerve fibers that do respond to sound synapse exclusively on IHC. Thus, the IHC in collaboration with the type I auditory nerve fibers provide the predominant source of auditory information to the central auditory pathway. The effects of selective IHC loss are currently poorly understood making *dwg/dwg* mice an especially useful model to determine what perceptual and electro-physiological changes occur when the auditory system is deprived of sensory information transmitted through the IHC-type I pathway.

In cases of carboplatin-induced destruction of IHC with retention of OHC, DPOAE and the CM remain normal. In contrast, the CAP generated by type I auditory nerve fibers is reduced in proportion to the amount of IHC loss [37, 39, 84–86]. While informative, the interpretations of the results are clouded by potential damage to the type I auditory nerve fibers and support cells [87–89]. Selective IHC loss has also been observed in *Slc19a2* mutant mice after low-thiamine challenge; however, most of these mice die by one month of age [90].

While most cases of hearing loss caused by genetic mutations, ototoxic drugs, acoustic trauma and aging are associated with a primary loss of OHC, hearing loss in *dwg/dwg* is only associated with IHC with retention of auditory nerve fibers. The time course and location of IHC loss in *dwg/dwg* mice can be compared to that seen in the few other animal models in which IHC loss is greater than OHC loss to determine if there are common pathological mechanisms.

#### *Bronx waltzer* (bv/bv) *mouse*

Mutant *bv/bv* mice are severely hearing impaired as a result of early IHC loss, which occurs between E17 and birth in the base of the cochlea and from birth to postnatal day 3 in the apex with little further loss beyond postnatal day 5 [91]. IHC loss, attributed to failure of differentiation, was caused by a mutation in the *Srrm4* gene, which affects tissue-specific alternative splicing [92]. This mechanism of congenital IHC loss is not likely involved in the age-related loss of IHC in *dwg/dwg* mice.

#### GLAST deficiency

Glutamate is the putative neurotransmitter released by IHC to activate type I auditory nerve fibers. To prevent excitotoxicity, excess glutamate is removed from the extracellular space by GLAST, a Na^+^-dependent glutamate-aspartate transporter. GLAST is heavily expressed in inner border cells and phalangeal cells surrounding IHC and is more heavily expressed in the middle and apical regions than the base of the cochlea [93]. In GLAST knockout mice (*Slc1a3^−/−^*), noise exposure increases glutamate levels in perilymph and exacerbates hearing loss [94]. Interestingly, IHC loss is greater in kanamycin-treated GLAST knockout mice than WT mice [95]; however, the mechanisms by which GLAST exacerbates IHC degeneration in aminoglycoside ototoxicity is not well understood. It was hypothesized that GLAST in supporting cells protects IHC by taking up excess glutamate, converting it to glutamine, which is neuroprotective, and then transferring it to IHC, similar to what occurs between glia and neurons [95–100].

#### Hypoxia and ischemia

Long-term hypoxia, known to induce excitotoxicity, causes more damage to IHC than OHC [101, 102]. Transient ischemia also causes IHC loss. Since ischemia-induced IHC loss was attenuated by DNQX, it was hypothesized that AMPA receptor induced excitotoxicity was a trigger for IHC loss [103]. Hypoxia, ischemia and oxygen-glucose deprivation also caused greater IHC loss than OHC loss in postnatal cochlear cultures [104]. Thus, selective IHC loss by hypoxia and ischemia may be related to the proposed mechanism of IHC loss by glutamate toxicity as described above for GLAST deficient mice. Administration of the free radical scavenger edaravone provided protection from ischemia-induced IHC loss [105] indicating that oxidative stress plays a role in this pathology.

#### Thiamine deficiency

Selective IHC loss occurs in *Slc19a2* knockout mice lacking the high affinity thiamine transporter [90]. Cochlear function remains normal in these knockout mice when treated with a high-thiamine diet, but if placed on a low-thiamine diet for 26 days, the mice develop IHC loss in the apical 60% of the cochlea. If thiamine deficiency continues for 36 days, IHC loss is accompanied by moderate OHC loss [90]. Thiamine-deficient *Slc19a2* mice develop 40-60 ABR threshold shifts, but smaller 10-20 dB DPOAE threshold shift, consistent with the greater loss of IHC than OHC [90]. The thiamine transporter is more abundant in IHC than OHC in normal mice [106], which may explain why IHC are more vulnerable than OHC in *Slc19a2* knockout mice. It is unclear why IHC loss is greater in the cochlear apex than the base in *Slc19a2* knockout mice, but one hypothesis is there is a base to apex gradient in this transporter. Oxidative stress has been shown to play a major role in the pathophysiology of thiamine deficiency [107] and thiamine deficiency was shown to reduce GSH levels and induce oxidative stress in brain mitochondria of mice [108].

#### Carboplatin-Induced IHC loss in chinchillas

While most platinum-based ototoxic drugs cause greater damage to OHC than IHC, an exception to this rule occurs in chinchillas treated with high doses of carboplatin, which destroys nearly all IHC without affecting the OHC or DPOAE [37, 39, 109]. GSH deficiency has been implicated in carboplatin ototoxicity in the chinchilla [47, 48], but the mechanism of selective IHC loss is still not fully understood. Carboplatin-induced IHC loss caused a reduction in CAP amplitude that was directly proportional to IHC loss. Surprisingly, when single unit recordings were made from carboplatin-treated chinchillas with partial IHC, the acoustically responsive auditory nerve fibers presumably contacting residual IHC, had low thresholds and sharp tuning equivalent to those obtained from normal control nerve fibers. These results suggest that the CAP amplitude reduction in this model was due to a reduction in the number of sound-activated fibers rather than a threshold elevation [37]. Interestingly, when behavioral hearing thresholds in quiet were measured in carboplatin-treated chinchillas, there was little change in behavioral thresholds until the IHC lesions exceeded 80% [110]. One interpretation of these results is that only a few functionally intact IHC connected to residual type I fibers are needed to detect a sound in quiet. In contrast to these results, we found that CAP thresholds were increased and amplitudes reduced in *dwg/dwg* mice in the region of IHC. Based on these results, we hypothesize that single auditory nerve thresholds would be elevated in *dwg/dwg* mice and that behavioral thresholds in *dwg/dwg* mice would also be elevated with moderate (30-80%) loss of IHC.

The preceding results from GLAST deficient mice, hypoxia, ischemia, thiamine deficiency and carboplatin toxicity as well as our results for GGT1 deficient *dwg/dwg* mice suggest that oxidative stress is a common mechanism leading to IHC loss. The degree of IHC loss and its selectivity, however, may be influenced by other factors such as the type and severity of the cellular insult and differences in genetic backgrounds. For example the selective effect of carboplatin toxicity on IHC loss occurs in chinchillas but not in other species examined [111]. The *Slc19a2^−^* [90] and Ggt1*^dwg^* mutations are on mouse strain backgrounds that do not have the *ahl* susceptibility allele of the *Cdh23* gene [112], which is associated with accelerated hearing loss and a primary loss of OHC [14]. If either of these mutations were examined in a strain that has the *Cdh23* susceptibility variant, the results likely would show OHC loss as well as IHC loss.

### Synopsis

The long-lived *dwg/dwg* mutant mouse with missing IHC, but intact OHC and auditory nerve fibers will provide auditory neuroscientists with an important new tool to study the functional consequences of IHC loss in the presence of OHC, for example the changes in auditory perception and electrophysiological properties of neurons in the central auditory pathway. Although the mechanisms that lead to the selective loss of IHC in *dwg/dwg* mice are poorly understood, our NAC supplementation studies indicate that oxidative stress or cysteine uptake are critically important factors; however, more in depth pharmacological and biochemical studies are needed to determine which signaling pathways trigger IHC death and why OHC are less vulnerable than IHC. IHC provide the only pathway by which acoustic information is transmitted to the central auditory pathway. Electrophysiological and animal behavioral studies could help unravel how neural activity relayed through the IHC/type I nerve fiber pathway affect hearing performance. Our results in GGT1 deficient mice and those of others [113] in thiamine deficient mice suggest that the majority of auditory nerve fibers can survive in the absence of IHC raising the question of which neurotrophic factors and support cells are responsible for their survival. IHC loss in *dwg/dwg* mice would reduce the afferent input to the central auditory pathway creating a condition of auditory deprivation. Recent studies suggest that the central auditory system increases its gain to compensate for the loss of cochlear input [85, 114]; whether a similar compensatory change occurs in *dwg/dwg* mice remains an intriguing, unanswered question.

## METHODS

### Mice

The mice used in this study were produced in the Research Animal Facility of The Jackson Laboratory (Bar Harbor, ME), which is accredited by the American Association for the Accreditation of Laboratory Animal Care. The dwarf gray (*Ggt1^dwg^*) mutation arose spontaneously on an inbred strain of unknown origin [24], which is available from the Jackson Laboratory (Stock #001743). We tested this strain for a *Cdh23* DNA variant that affects ARHL in many inbred mouse strains [112] and found it to be homozygous for the *Cdh23^753G^* allele, which confers AHL resistance, thus eliminating potential confounding contributions of this gene to hearing loss assessments of *dwg/dwg* mutant mice. Breeding, genotyping and auditory brainstem response (ABR) hearing tests were conducted at the Jackson Laboratory and the cochleae from these mice were fixed and shipped to the University at Buffalo for histological evaluation. Some live mice between 3 and 9 months of age were shipped to the University at Buffalo for testing of distortion product otoacoustic emissions (DPOAE) and measurements of the cochlear microphonic (CM) potential and compound action potential (CAP) followed by histological analysis of the cochlea.

### Genotyping

Homozygous *dwg/dwg* mice are viable but do not breed; therefore, the mutation is maintained by heterozygous matings, and genotypes of progeny are identified by PCR analysis. The *dwg* mutation is a 13 bp deletion within exon 7 of the gamma-glutamyl-transferase 1 (*Ggt1*) gene [20]. We used forward primer CTGATTGAGCATCCGATGAG and reverse primer CCTCAGCAGGGCTAGAGAGA, which flank the *dwg* deletion, to amplify genomic DNA extracted from tail tips of mice. The resulting PCR products, 136 bp from the WT allele and 123 bp from the *dwg* allele, were resolved on a 3% agarose gel and visualized by ethidium bromide staining.

### ABR

ABR thresholds were measured at 8, 16 and 32 kHz in a sound attenuating chamber using the SmartEP auditory evoked potential diagnostic system from Intelligent Hearing Systems (IHS, Miami, FL) as described previously [14, 15, 115, 116]. Briefly, mice were anesthetized with tribromoethanol (0.2 ml of 20 mg/ml stock per 10 g of body weight, i.p.) and placed on a temperature controlled heating pad to maintain body temperature at 37°C. Subdermal electrodes placed at the vertex and behind the ipsilateral and contralateral ears were used to record the ABR to tone-bursts (3 ms duration. 1.5 ms cosine-gated rise/fall time) were amplified, filtered (100-3000 Hz) and averaged (25 kHz sampling rate, 10 ms analysis window). Stimulus intensity was initially decreased in 10 dB steps until the response began to disappear and then lowered in 5 dB steps; ABR threshold was defined as the lowest intensity at which an ABR response could be reliably obtained. Our average ABR thresholds for normal hearing mice are about 40, 20 and 45 dB SPL for 8, 16 and 32 kHz respectively [115].

### DPOAE

DPOAE input/output (I/O) functions were measured as described previously [117–119]. Mice were anesthetized with ketamine (80 mg/kg, i.p.) and xylazine (6 mg/kg, i.p.). The amplitude of 2F1-F2 was measured in a sound attenuating chamber (Smart Distortion Product Otoacoustic Emission System, Intelligent Hearing System) using an earpiece containing a microphone (Etymotics 10B+) and two sound delivery tubes connected to two high frequency transducers used to deliver primary tones, F1 and F2 to the ear canal at an F2/F1 ratio or 1.2. The microphone output was measured using system software. DPOAE I/O functions were measured at F2 frequencies of 13, 16, 24 and 30 kHz and with the level of F2 set 10 dB lower than F1. The intensity of F1 was varied from 10 to 60 dB SPL in 5-dB steps. The DPOAE spectrum was averaged over 32 sweeps.

### CM and CAP

The CM was recorded as previously described [120, 121]. Mice were anesthetized with ketamine (80 mg/kg, i.p.) and xylazine (6 mg/kg, i.p.), placed on temperature controlled heating pad set to 37°C and the cochlea surgically opened to expose the round window membrane. A small, silver ball electrode was placed on the round window for recording the CM and CAP. A silver chloride reference electrode was placed beneath the neck skin. Tone bursts (4, 6, 8, 12, 16, 20, 24, 35 and 40 kHz, 10 ms duration, 1 ms rise/fall time, cosine gated, fixed starting phase) were generated using a real time processor (RP2.1, system3, TDT, Gainesville, FL), amplified, attenuated (TDT PA5) and delivered to a speaker (ACO half-inch 7013 microphone, driven in reverse) located in a speculum-like housing. The sound delivery system was inserted into the ear canal near the tympanic membrane. The acoustic output was calibrated with an eighth inch microphone (4138 B&K) using a coupler approximating the volume of the ear canal. Cochlear responses were amplified with a Grass A.C. preamplifier (Model P15, 1000X, 0.1 Hz–50 kHz) and digitized (20 ms time window, 100 kHz sampling rate) and averaged (50X) with a TDT RP2.1 real time processor using custom data acquisition and analysis software (MATLAB 6.1). The CAP was obtained by low-pass filtering at 3 kHz. Mean CAP I/O functions (amplitudes vs. intensity) were generated for WT (*+/+*) and experimental mice (*+/dwg* and *dwg/dwg*) and used to estimate the amount of threshold shift (TS). The CM was obtained by high-pass filtering at a frequency1 kHz below the stimulus frequency.

### Cochleograms and cochlear histology

After completing the ABR measurements at 1, 3, 6, and 9 month of age at The Jackson Laboratory the inner ears were dissected out, immersed in 4% paraformaldehyde and shipped to the University at Buffalo for analyses of the cochlea and vestibular system. Our procedures for preparing cochleograms showing the percentage of missing inner hair cells (IHC) and outer hair cells (OHC) as a function of percent distance from the apex have been described in detail previously [14, 15, 122]. Mice evaluated by ABR at the Jackson Lab were euthanized by CO_2_ asphyxiation and decapitated. The temporal bones were removed, immersed in 4% paraformaldehyde, and shipped to the University at Buffalo for analysis. Cochleae were stained with Ehrlich's hematoxylin solution, the organ of Corti dissected out as a flat surface preparation, mounted in glycerin on glass slides and coverslipped. A person, blind to the results, dissected the cochleae and prepared the surface preparation. A second person blind to the experimental conditions counted the hair cells using a light microscope (Zeiss Standard, 400X magnification). By raising and lowering the focal plane, the investigator can determine if the hair cell nucleus, cuticular plate and stereocilia bundle were present. A hair cell was counted as present if both the cuticular plated and nucleus were clearly visible and considered missing if either were absent. OHC and IHC were counted along successive 0.12-0.24 mm intervals from the apex to the base. Using lab norms and custom software, the percentage of missing IHC and OHC were determined for each animal and a cochleogram was constructed showing the percentage of missing OHC and IHC as a function of percent distance from the apex of the cochlea. Position in the cochlea was related to frequency using a mouse tonotopic map [33]. In some cases, the cochlear surface preparations were photographed with a digital camera (SPOT Insight, Diagnostic Instruments Inc.) attached to a Zeiss Axioskop microscope, processed with imaging software (SPOT Software, version 4.6) and Adobe Photoshop 5.5.

To evaluate the condition of the cochlea and vestibular sensory epithelium in more detail, some inner ears were embedded in plastic using procedures described in our earlier publications [32, 122, 123]. Following fixation, inner ears were decalcified (Decal, Baxter Scientific Products), rinsed in phosphate buffered saline, dehydrated through a graded series of EtOH and then embedded in Epon 812 (Electron Microscopy Sciences). Sections were cut parallel to the modiolus of the cochlea at a thickness of three μm on an ultramicrotome, stained with 0.5% toluidine blue, mounted on glass slides, examined with a Zeiss microscope (Axioskop) and photographed with a digital camera as above. Sections (3 μm) were also taken from the utricle, saccule and crista ampullaris following similar procedures.

### NAC supplementation

To determine if NAC supplementation would prevent ARHL and cochlear pathology during NAC treatment and after NAC supplementation was discontinued, one group *of dwg/dwg* mice was supplied ad libitum with NAC (#A7250, Sigma, St. Louis, MO) dissolved in drinking water (10 g/liter) from 3 weeks of age until 6 months of age. Water bottles containing NAC were changed weekly. NAC supplementation was discontinued at 6 months of age, but mice were evaluated again at 9 months of age to determine if the otoprotective effects of the 6-month treatment would be maintained or lost. To determine if NAC supplementation would affect developmental growth, treated and untreated mice were periodically weighed to the nearest 0.1 gram on a lab scale. ABR thresholds were measured in NAC-treated *dwg/dwg* mice at 3, 6 and 9 months of age and compared to ABR thresholds of untreated *dwg/dwg* and WT mice of the same age. Afterwards the cochleae were harvested to assess the degree of hair cell loss.

### Animal care

All procedures involving the use of experimental mice were approved by the Institutional Animal Care and Use Committees of The Jackson Laboratory and the University at Buffalo.
